# Sustainable biosurfactant produced by *Serratia marcescens* UCP 1549 and its suitability for agricultural and marine bioremediation applications

**DOI:** 10.1186/s12934-018-1046-0

**Published:** 2019-01-04

**Authors:** Hélvia W. C. Araújo, Rosileide F. S. Andrade, Dayana Montero-Rodríguez, Daylin Rubio-Ribeaux, Carlos A. Alves da Silva, Galba M. Campos-Takaki

**Affiliations:** 1 Chemistry Department, State University of Paraíba, Campina Grande, PB 58429-500 Brazil; 2 Nucleus of Research in Environmental Sciences and Biotechnology, Catholic University of Pernambuco, Recife, PE 50050-590 Brazil; 3 Center of Biosciences, Federal University of Pernambuco, Recife, PE 50670-420 Brazil

**Keywords:** Biosurfactant, *Serratia marcescens*, Cassava flour wastewater, Seed germination, Oil removing

## Abstract

**Background:**

Biosurfactants are surface-active agents produced by microorganisms that have higher efficiency and stability, lower toxicity and higher biocompatibility and biodegradability than chemical surfactants. Despite its properties and potential application in a wide range of environmental and industrial processes, biosurfactants are still not cost-competitive when compared to their synthetic counterparts. Cost effective technologies and renewable raw substrates as agro-industrial and regional waste from northeast of Brazil as cassava flour wastewater, supplemented with lactose and corn oil are mainly the chemically media for growing microorganism and in turn the production of the biosurfactant of quality. This study aimed to obtained biosurfactant by *Serratia marcescens* UCP 1549 containing cassava flour wastewater (CWW), by application of a full-factorial design, as sustainable practices in puts the production process in promising formulation medium. The characterization of the biomolecule was carried out, as well as the determination of its stability and toxicity for cabbage seeds. In addition, its ability to stimulate seed germination for agriculture application and oil spill bioremediation were investigated.

**Results:**

*Serratia marcescens* showed higher reduction of surface tension (25.92 mN/m) in the new medium containing 0.2% lactose, 6% cassava flour wastewater and 5% corn waste oil, after 72 h of fermentation at 28 °C and 150 rpm. The substrate cassava flour wastewater showed a promising source of nutrients for biosurfactant production. The isolate biosurfactant exhibited a CMC of 1.5% (w/v) and showed an anionic and polymeric structure, confirmed by infrared spectra. The biomolecule demonstrated high stability under different temperatures, salinity and pH values and non-toxicity against to cabbage seeds. Thus, exploring biosurfactant their potential role in seeds germinations and the promotion and agricultural applications was investigated. In addition, the effectiveness of biosurfactant for removal burned motor oil adsorbed in sand was verified.

**Conclusions:**

The use of medium containing CWW not only reduces the cost of process of biosurfactant production, but also the environmental pollution due to the inappropriate disposal of this residue. This fact, added to the high stability and non-toxicity of the biosurfactant produced by *S. marcescens* UCP 1549, confirms its high environmental compatibility, make it a sustainable biocompound that can be replace chemical surfactants in diverse industries. In addition, the effectiveness of biosurfactant for stimulate seed germination and removing burned motor oil from sand, suggests its suitability for agriculture and bioremediation applications.

**Electronic supplementary material:**

The online version of this article (10.1186/s12934-018-1046-0) contains supplementary material, which is available to authorized users.

## Background

Surfactants are amphipathic molecules with hydrophobic (nonpolar) and hydrophilic (polar) moieties that reduce surface and interfacial tension between liquid. According to their origin, they can be classified as chemical surfactants (derived from petroleum) or biosurfactants (microbial origin) [1, 2].

Biosurfactants are secondary metabolites produced by various microorganisms (bacteria, yeasts and filamentous fungi), and they are classified according to their chemical composition and microbial origin. Producing microorganisms are distributed in various genres, mostly bacteria; some examples are *Pseudomonas aeruginosa*, *Bacillus subtilis* and *Lactobacillus* sp. [3, 4]. The major classes of biosurfactants include glycolipids, lipopeptides, phospholipids, fatty acids, lipoproteins and polymeric and particulates compounds [5, 6].

In recent decades, the interest in biosurfactants has increased due to their advantages over their synthetic counterparts, including better environmental compatibility, production from renewable waste substrates, maintaining activity at harsh environmental conditions, lower or no environmental toxicity. These properties makes feasible to utilize them for numerous environmental, food, pharmaceutical, medical, cleaning and other industrial application purposes [7–10].

However, widespread industrial applications of biosurfactants are quite limited, mainly due to cost-competitiveness. Chemical surfactants are still comparatively cheaper than biosurfactants [3, 11, 12]. However, the characteristics of the non-biodegradability, ability to accumulate and toxicity of some of the chemical petroleum based product to the environment suggest to find replacement surfactants to the chemically synthesized compounds with ecofriendly products as biosurfactant or bioemulsifier [10–12]. Therefore, different strategies are currently explored to improve biosurfactant production economics. Among them, the use of low-cost raw materials such as agro-industrial wastes is a sustainable alternative since the utilization of these residues contributes to the reduction of environmental pollution [6, 8, 13]. A characteristic wastewater from industrial cassava processing has a huge adverse impact on the environment by large amount of cyanogenic glucosides (linamarin and lotaustralin) is produced [14]. Brazil is one of the largest global producers of cassava (*Manihot esculenta* Crantz) liquid waste (“manipueira”) whose processing to produce flour and starch gives rise to about 250–300 L wastewater per ton of processed cassava, mainly composed of organic matter and nutrients [15, 16].

In addition, optimization of culture medium, growth conditions and process parameters by using statistical models can also significantly increase the yield and reduce the overall cost [17, 18].

*Serratia marcescens*, a facultative bacterium belonging to the Enterobacteriaceae family, produces biosurfactant such as serrawetins that present enormous potential of application in pharmaceutical industry [19, 20]. Over the last years, several studies have reported the production of biosurfactants by *S. marcescens* strains using a variety of hydrophilic and hydrophobic substrates [21–24]. However, researches focusing on the use of agro-industrial waste are still few and they have been carried out by our research group [8, 25]. In addition, the toxicity of this secondary metabolite has only been tested in mice [26], and considering its potential for environmental application, further studies are needed.

In this sense, this work aimed to the improved production of biosurfactant by *S. marcescens* UCP 1549 in low-cost fermentative medium containing cassava flour wastewater (CWW). The effect of the medium components was investigated by full factorial design (FFD) and the best condition was selected and used for kinetics of microbial growth and biosurfactant production. The isolation and characterization of biosurfactant were carried out, as well as studies of its stability, phytotocity and application in removal burned engine oil.

## Results and discussion

### Biosurfactant production

In this study, the production of biosurfactant by *S. marcescens* UCP 1549 was improved using the 2^3^ FFD proposed in Table 1, in order to determine the relationship and influence between input and output variables on the process efficiency. The following response equation (Eq. 1) was used to correlate the dependent and independent variables:

1 $$Y = b_{0} + b_{1} x_{1} + b_{2} x_{2} + b_{3} x_{3} + b_{12} x_{1} x_{2} + b_{13} x_{1} x_{3} + b_{23} x_{2} x_{3} + b_{123} x_{1} x_{2} x_{3}$$

where *Y* is the response variable or surface tension, *b*_0_ is a constant; *b*_1_, *b*_2_ and *b*_3_ are regression coefficients for the linear effects and *b*_12_, *b*_13_, *b*_23_ and *b*_123_ are interaction coefficients.

**Table 1:** Variables levels used in the full-factorial design for biosurfactant production by *Serratia marcescens* UCP 1549

Variables	Levels		
	Low (−1)	Central (0)	High (+1)
Lactose (% m/v)	0.2	0.6	1.0
Cassava flour wastewater (% v/v)	1.0	3.5	6.0
Corn oil (% v/v)	5	6.25	7.5

To analyze the mathematical models, adjustments to the points were made by nonlinear regression methods and Table 2 shows the experimental and predicted values of the model to obtain the lower surface tension.

**Table 2:** Full-factorial design for the biosurfactant production by *Serratia marcescens* UCP 1549 after 72 h

Assay	Lactose (%)	Cassava flour wastewater (%)	Corn oil (%)	Surface tension (mN/m)	
				Experimental	Predicted
1	− 1	− 1	− 1	29.33	28.77
2	1	− 1	− 1	42.29	41.73
3	− 1	1	− 1	25.92	25.36
4	1	1	− 1	28.34	27.78
5	− 1	− 1	1	29.29	28.73
6	1	− 1	1	34.63	34.07
7	− 1	1	1	26.78	26.22
8	1	1	1	29.07	28.51
9	0	0	0	29.03	30.14
10	0	0	0	29.33	30.14
11	0	0	0	28.82	30.14
12	0	0	0	28.89	30.14

The regression coefficient values, standard deviation, *t*_*exp*_ and significance level are represented in Table 3. As can be seen all regression and interaction coefficients were significant, with confidence level 95% (p ≤ 0.05), determined by the analysis of variance (ANOVA).

**Table 3:** Estimated regression coefficients and corresponding *t*_*exp*_ and significance levels for the surface tension

Coefficient	Value	Standard deviation	*t*_*exp*_	*p*
*b*_*0*_	30.1433	0.0652	462.2643	0.0000
*b*_*1*_	2.8763	0.0799	36.0147	0.0001
*b*_*2*_	− 3.1786	0.0799	− 39.8025	0.0000
*b*_*3*_	− 0.7638	0.0799	− 9.5632	0.0024
*b*_*12*_	− 1.6988	0.0799	− 21.2708	0.0002
*b*_*13*_	− 0.9688	0.0799	− 12.1301	0.0012
*b*_*23*_	1.1613	0.0799	14.5405	0.0007
*b*_*123*_	0.9363	0.0799	11.7232	0.0013

The application of response surface methodology (RSM), offers, on the basis of parameter estimation (Table 3), the following empirical relationship (Eq. 2) between the surface tension (Y) and independent variables studied.

2 $$Y = 30.1433 + 2.8763x_{1} - 3.1786x_{2} - 0.7638x_{3} - 1.6988x_{1} x_{2} - 0.9688x_{1} x_{3} + 1.1613x_{2} x_{3} + 0.9363x_{1} x_{2} x_{3}$$

**Fig. 1:**
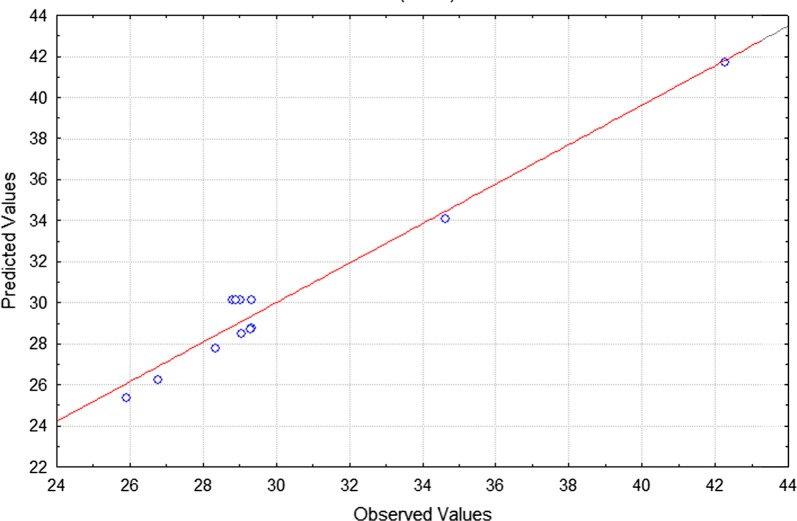
Observed values versus predicted values by model for the answer surface tension

The predicted versus the actual plot for surface tension determined by the model equation demonstrated that observed values were distributed near the straight line (Fig. 1), which indicates that such values were close to the predicted values. The ANOVA showed that the regression model had a high coefficient of determination (R^2^ = 0.963), indicating that 96.3% of the variation in the process efficiency was explained by the independent variables and that only 3.7% was not explained by the model. Reproducibility of the experimental data was confirmed by the low pure error (0.051) and value of the adjusted determination coefficient (Adj. R^2^ = 0.897). Hence, the model proved to be suitable for the prediction of biosurfactant production under the experimental conditions.

**Fig. 2:**
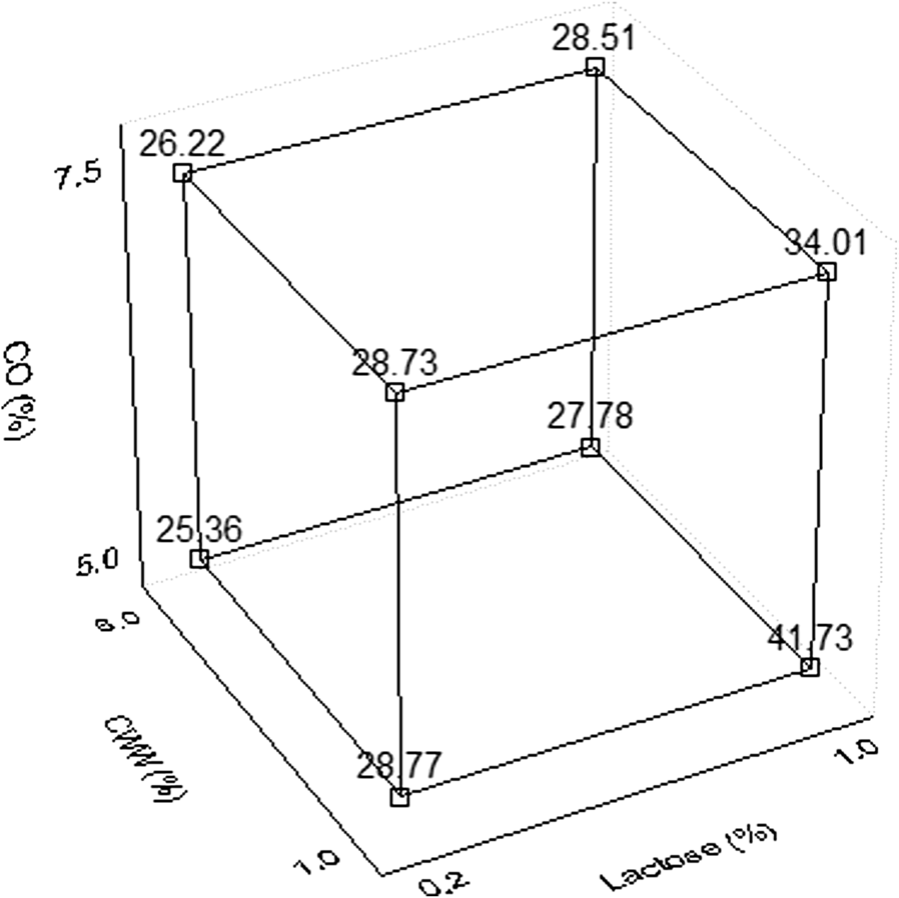
Cubic plot of the surface tension values estimated from the full-factorial design of Table 2

The reduction in surface tension is commonly used as a primary criterion for selecting biosurfactant-producing microorganisms [2, 27, 28]. In this study, surface tension was used as response variable, and the effects of independent variables (lactose, CWW and CO concentrations) as well as the interactions of them, were analyzed by estimated effects represented in Table 3 and Fig. 2. It can be concluded that although the three variables were significant, the increase of all of them led to increment of surface tension. However, the increment of CWW and diminution of CO and lactose allowed the lower value of surface tension, as verified in Table 2, in condition 3 of the FFD.

Previously, Araújo et al. [25] used the same substrates and FFD for the production of biosurfactant by *S. marcescens* UCP 1549, but in static conditions, and they found the best results in condition 8, with reduction on surface tension to 30.60 mN/m. Highest tensioactive activity detected in current study in flasks with agitation can be linked to the physiological function of the biosurfactants. It has been suggested that their production can increase the solubility of hydrophobic substrates in water and, consequently, facilitate the transport of nutrients to microorganisms [3, 29]. Therefore, the shear stress caused by agitation can induce larger biosurfactant excretion since the contact between the organic phase drops, dispersed in water, and the microorganisms becomes more difficult [30]. In addition, several researchers have indicated that an increase in agitation speed favored the production of biosurfactant, since that it influences the mass transfer efficiency of both oxygen molecules and medium components [31, 32].

In other hand, it was ratified the suitability of CWW as low-cost substrate for biosurfactant production. Commonly, it is used as nitrogen source, because it has a high presence of nitrogen in its composition [8, 25, 33, 34] (see Additional file 1: Table S1). Earlier, Ferraz et al. [33] showed the ability of the biosurfactant produced by *S. marcescens* to reduce water surface tension of 72–28 mN/m using CWW as substrate. Similarly, this residue was used for biosurfactant production by *Bacillus* sp. and results showed a reduction in surface tension of 59–26 mN/m [34]. The use of CWW can contribute considerably to the diminution of cost production of biosurfactants, since it is estimated that raw materials represent 30% of the total costs of bioprocess [1, 13, 35].

### Growth kinetics and biosurfactant production

**Fig. 3:**
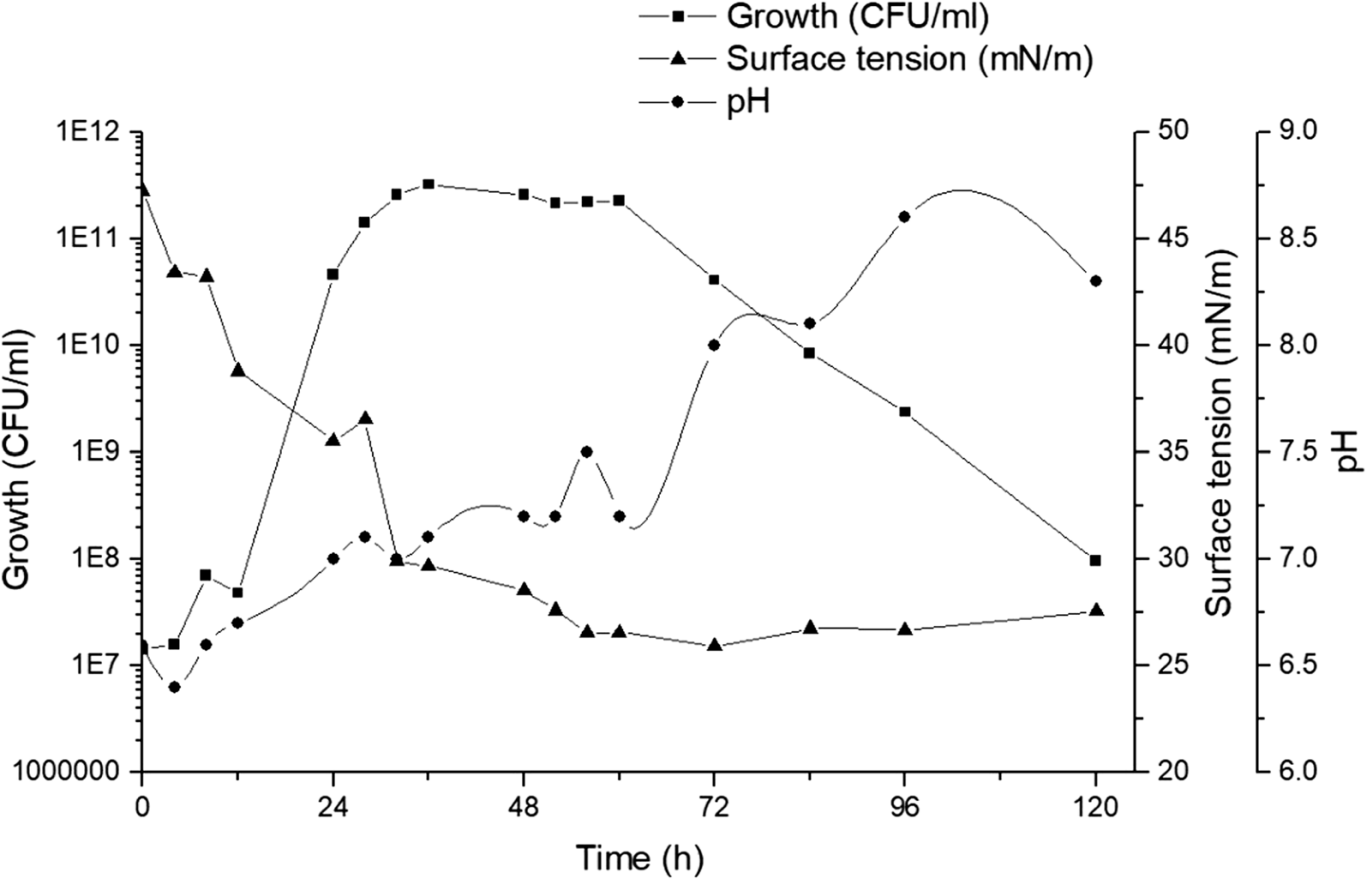
Kinetic profiles of growth, surface tension and pH of *Serratia marcescens* UCP 1549. The strain was grown in medium containing 0.2% lactose, 6% CWW and 5% CO during 120 h at 150 rpm and 28 °C

After the selection of the medium with higher reduction of surface tension, the microbial growth kinetics and biosurfactant production were monitored during 120 h. Figure 3 shows the production kinetics of biosurfactant produced by *S. marcescens* UCP 1549 in medium containing 0.2% lactose, 6% CWW and 5% CO.

As can be observed, the exponential phase of microorganism occurred in the first 24 h, after a very short phase of adaptation to physico-chemical composition of medium. Simultaneously, the surface tension began to decrease, indicating that the new substrates promote the biosynthesis of essential compounds to the microbial growth and the production of biomolecules with surface active properties. The start of growth stationary phase occurred at 36 h when the value of surface tension was lower to 30 mN/m. Similar profile was observed when *S. marcescens* UCP 1549 was grown in LB medium (see Additional file 1: Figure S1). However, the maximum production of biosurfactant was detected at 72 h, with lower value of surface tension (25.9 mN/m).

According to the literature, the bacterial biosurfactants are more effective in reduce surface tension. Particularly, the bacterium *Pseudomonas aeruginosa* have been the most studied microorganism to produce potent biosurfactant, with the ability to reduce surface tension to values around 28–27 mN/m [36–38]. However, lastly other microorganisms have been explored in this sense, such as *S. marcescens* strains [21–26]. Pruthi and Cameotra [39] obtained a reduction of 68–27 mN/m in mineral medium containing 2% glucose by *S. marcescens*. Recently, Rosas-Galván et al. [24] informed a reduction to 26.5 mN/m of biosurfactant produced by *S. marcescens* SM3.

### Stability of biosurfactant

**Fig. 4:**
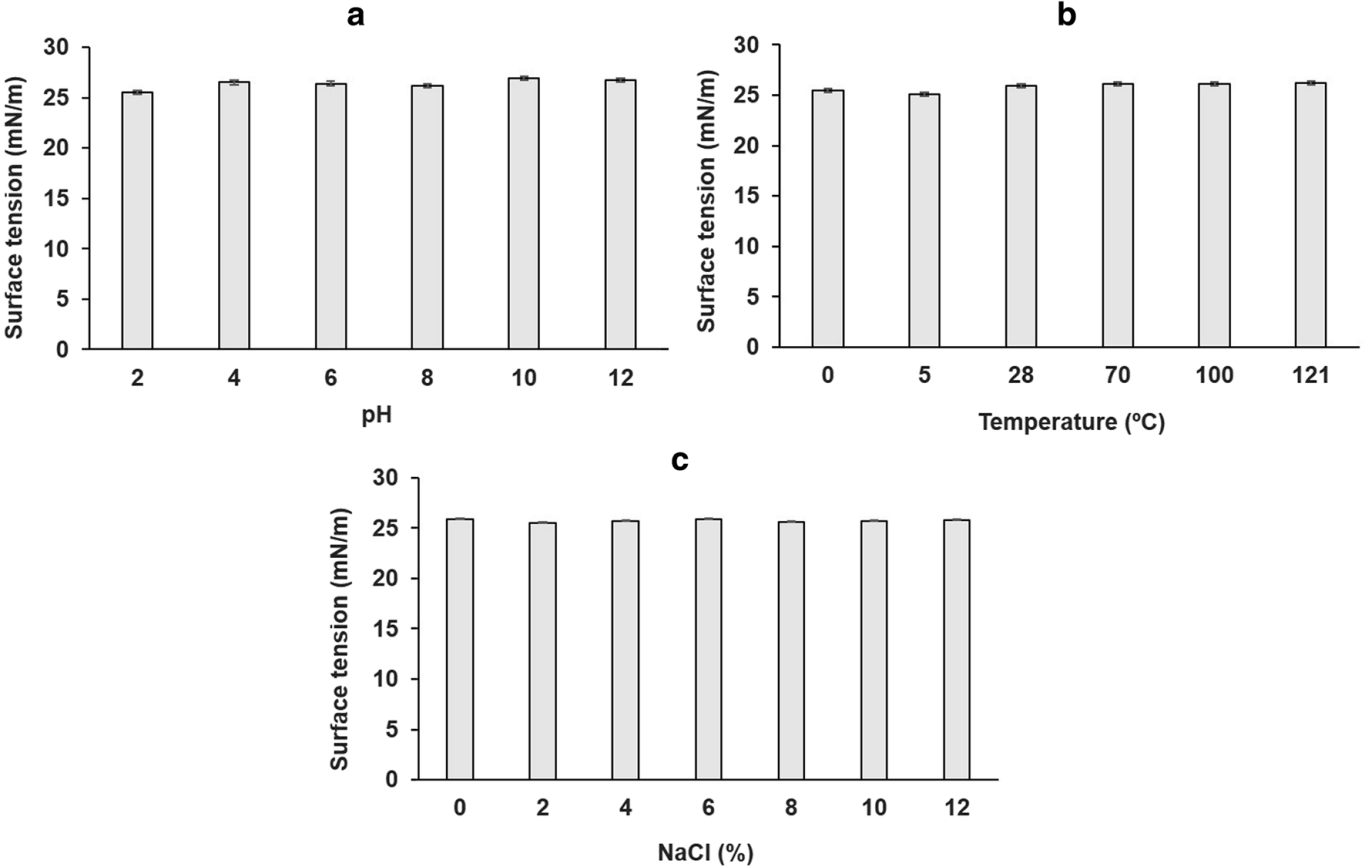
Stability of surface tension of biosurfactant produced by *Serratia marcescens* UCP 1549. Influence of pH (**a**), temperature (**b**) and sodium chloride concentrations (**c**) on surface tension of biosurfactant produced by *S. marcescens* UCP 1549 after 72 h of fermentation

The suitability of biosurfactants for application in diverse industrial areas depends on its stability against extreme or varying conditions of temperature, pH and salinity. The suitability of biosurfactants for application in diverse industrial areas depends on its stability against extreme or varying conditions of temperature, pH and salinity [40]. Figure 4 illustrates the effects of temperature, pH and NaCl concentration on the surface tension of the biosurfactant produced by *S. marcescens* UCP 1549.

The results showed that the surface tension remained practically uniform (25.1–26.8 mN/m) at all conditions tested, evidencing the stability of the biosurfactant produced by *S. marcescens* UCP 1549 against extreme values of pH, temperature and NaCl concentration. Previously, these parameters had no appreciable effects on the activity of the biosurfactant produced by *S. marcescens* UCP 1549 in CWW and waste soybean oil [8]. Similarly, biosurfactants produced by other *S. marcescens* strains have indicated stability in a wide range of pH, temperature and salinity [22, 26], make them potential candidates for environmental or industrial processes in extreme conditions [3, 41].

### CMC of biosurfactant

**Fig. 5:**
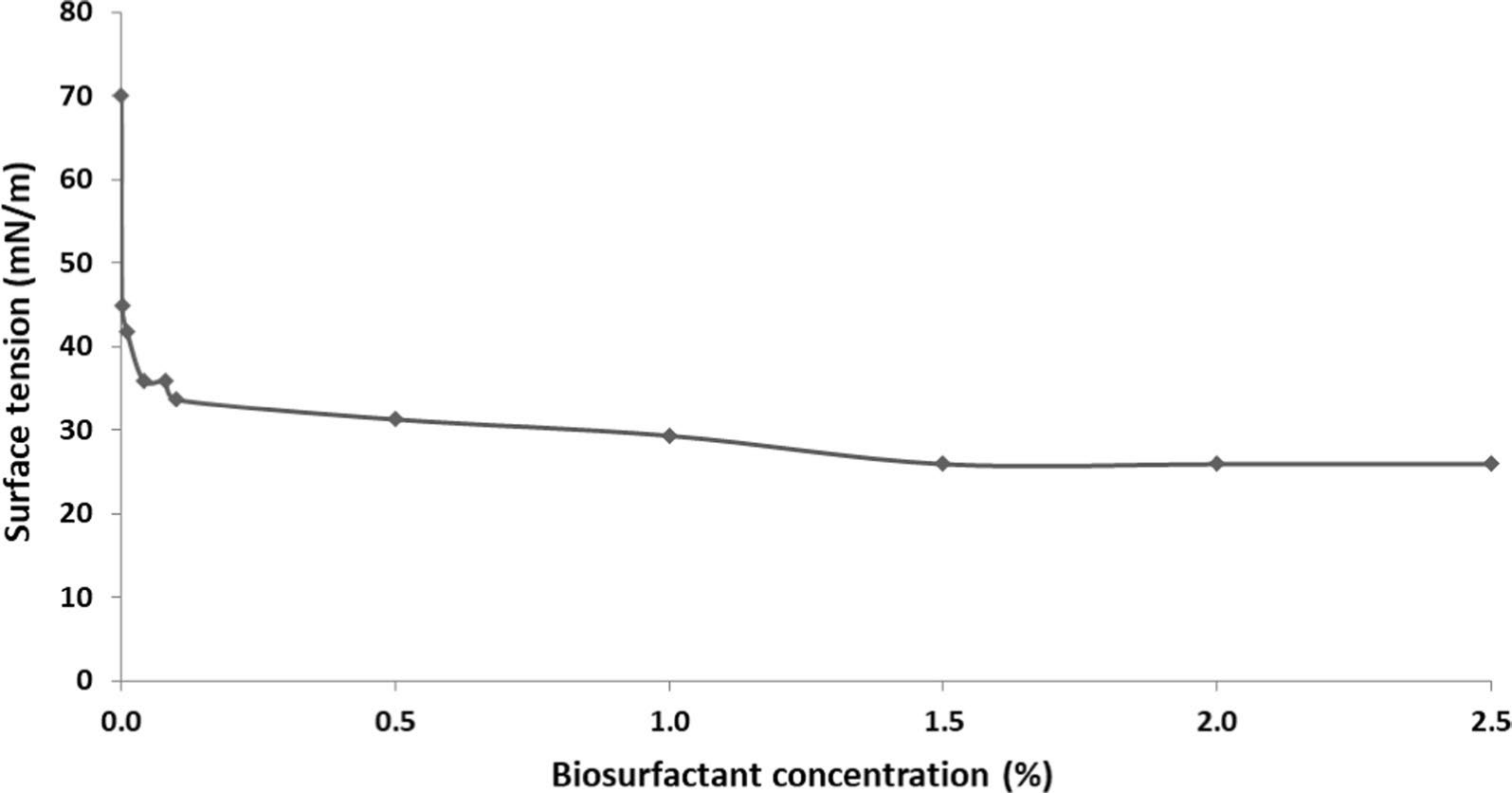
Critical micelle concentration of the biosurfactant produced by *Serratia marcescens* UCP 1549. Surface tension versus concentration of isolated biosurfactant produced by *S. marcescens* UCP 1549 after 72 h of fermentation

The critical micelle concentration (CMC) is an important physiochemical parameter used to evaluate biosurfactant activity, which indicates the minimum concentration of biosurfactant necessary to achieve the lowest stable surface tension [37, 42]. Efficient surfactants have low CMC values, i.e., less surfactant is required to decrease surface tension [13, 43]. As shown in Fig. 5, biosurfactant produced by *S. marcescens* UCP 1549 exhibited a CMC of 1.5%, with surface tension of 25.92 mN/m.

This CMC was higher compared to others biosurfactants produced by *S. marcescens* strains but the reduction of surface tension was noticeably greater for biosurfactant produced by *S. marcescens* UCP 1549. Anyanwu et al. [26] reported the production of biosurfactant by *S. marcescens* NSK-1, which exhibited a CMC of 29 mg/L (0.03%) and reducing the surface tension of distilled water from 72 to 38 mN/m. Previously, *S. marcescens* UCP 1549 exhibited CMC of 2.5% with surface tension of 33.8 mN/m [44].

### Characterization of biosurfactant

The biosurfactant produced by *S. marcescens* UCP 1549 was isolated and appeared as a light brown precipitated soluble in water. The chemical composition analyses revealed the presence of 43% lipids, 32% proteins and 11% carbohydrates, suggesting its polymeric nature.

**Fig. 6:**
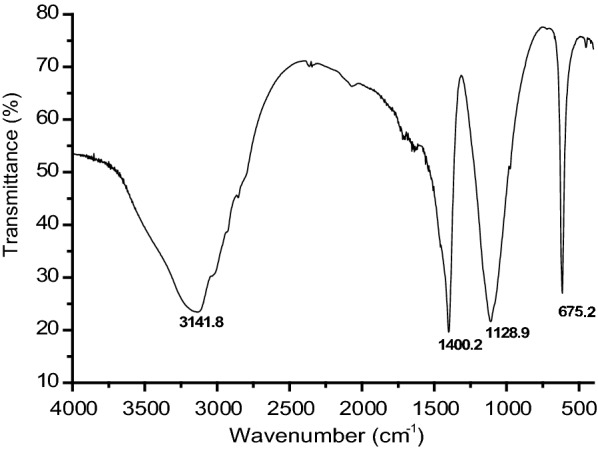
Fourier transform-infrared (FT-IR) spectra of the biosurfactant produced by *Serratia marcescens* UCP 1549

Biosurfactants are generally macromolecules and it is not easy to determine their chemical structure, especially when they are produced from industrial wastes. However, the determination of the structural formula of these compounds is fundamental to study and better understand their properties, as well as to propose the most appropriate field of its application [3, 45]. Fourier transform infrared (FT-IR) is a powerful tool that has been extensively used to study the different forms of biosurfactants [2, 13, 46–48]. Partially purified biosurfactant produced by *S. marcescens* UCP 1549 was subjected to FT-IR and the spectra is shown in Fig. 6.

The FT-IR spectrum showed a significant absorbance peak at 3141 cm^−1^ ranging from 2700 to 3700 cm^−1^. This feature typically is attributed to the presence of carbon and amino groups and caused due to stretching vibrations of C–H and N–H bonds, present in the peptide moiety. A sharp band at 1400.2 cm^−1^ correspond to C–H stretching mode, suggesting the presence of aliphatic chain of fatty acids, whereas the absorption peak, located at 1128.9 cm^−1^ showed the presence of ester carbonyl groups (–CO bond), indicating the ester linkage between the polysaccharides-forming monomers. At last a sharp peak at 675.2 cm^−1^ represented –CH bending of alkenes [49, 50].

The source of carbon and nitrogen used in the biosurfactant production is considered one of the most important parameters that influences in biosurfactant composition. In this work, *S. marcescens* UCP 1549 was able to use lactose and corn oil, probably as carbon source because they are carbohydrates and fatty acids respectively, proving the ability of microorganisms to utilize some carbohydrates and oils as carbon source in the induction of production of secondary metabolites [3, 51]. Cassava flour wastewater was used as nitrogen source, because it has a high presence of nitrogen in its composition [34, 52]. Studies show that to obtain high production of biopolymer, one should better define the relationship between sources of carbon and nitrogen in the medium. The balanced medium can contain ten times more carbon than nitrogen ensuring high protein content, while a ratio greater than 50:1 promotes the accumulation of extracellular secondary metabolites [1].

In other hand, the zeta potential determines the ionic charge of the particle, which serves to predict and control the stability of colloidal suspensions and emulsions. Higher values of zeta potential indicate good stability of the suspension, due to the repulsion between hydrophilic particles, according to the literature [53, 54]. According to the analyses using a Zeta Potential Meta 3.0+, the biosurfactant produced by *S. marcescens* showed an anionic character (Pz = − 20.13 mV). Other biosurfactants produced by *Serratia* sp. also display an anionic character [39, 55, 56]. The most commercially used surfactants are anionic, with extensive application in household cleaners and cosmetics [13, 57].

### Phytotoxicity of biosurfactant

**Fig. 7:**
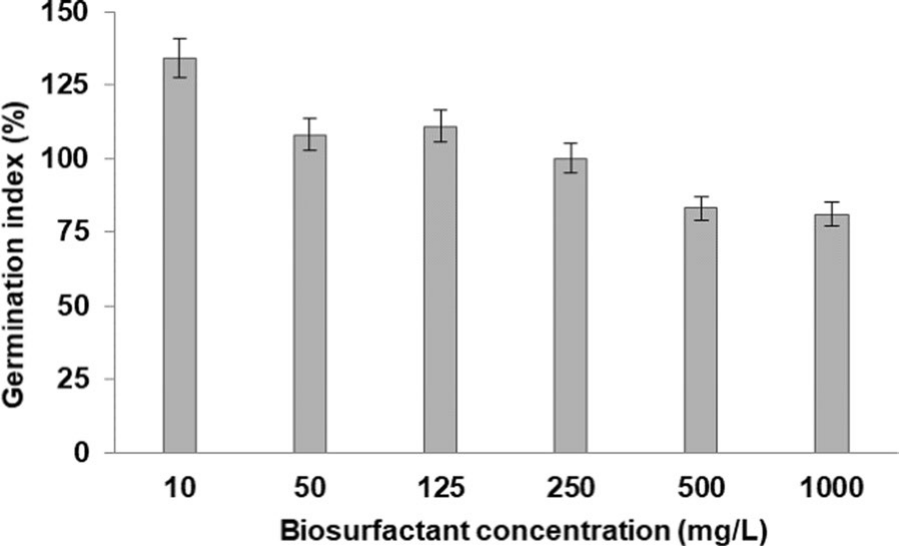
Phytotoxicity of biosurfactant produced by *Serratia marcescens* UCP 1549 on seeds of cabbage (*Brassica oleracea*)

The use of plants in toxicity tests offers several advantages, among them low maintenance cost and rapid results, with a special benefit assessment of the potential eco-toxic compounds in terrestrial environments [58, 59]. In this study, the germination index (GI), which combines measures of relative seed germination and relative root elongation [2, 43, 60], has been used to evaluate the toxicity of the biosurfactant on cabbage (*B. oleracea*), and the results are represented in Fig. 7.

Considering that a GI value of 80% has been used as an indicator of the disappearance of phytotoxicity [51, 62], the results obtained here indicated that the biosurfactant solutions tested did not show inhibitory effects on seed germination and root elongation of cabbage, after 5 days of incubation. GI of 134,108, 111, 100, 83 and 81 were found for biosurfactant solutions of 10, 50, 125, 250, 500 and 1000 mg/L, respectively. Similar results were observed by Silva et al. [60] who investigated the phytotoxic potential of biosurfactant from *P. aeruginosa* UCP 0992 cultivated in glycerol and found that the compound was innocuous with regard to cabbage. Recently, biosurfactant produced by *Streptomyces* sp. DPUA1559 in mineral medium containing 1% residual frying soybean oil not display inhibitory effects for seeds of cabbage, at concentrations of 1, 5 and 50 mg/mL [2]. Several researches have reported the phytotoxicity studies of biosurfactants [47, 63–65], but to our knowledge this is the first report regarding toxicity of biosurfactant from *S. marcescens*.

Interestingly, in this study it was possible to observe the presence of secondary roots and leaves on cabbage seeds, for all the biosurfactant solutions tested, suggesting that the biosurfactant produced by *S. marcescens* UCP 1549 showed a positive effect on the germination and growth of seeds. Rubio-Ribeaux et al. [47], Krawczyńska et al. [66], and Alsohim et al. [67] reported similar findings, when detected the positive effect of biosurfactants in seedling development. According to Kerbauy [68], the process of germination begins when the seed coat allows the entrance of water in the seed, which activates metabolism and leads to growth of the embryonic axis. For this process to take place, it is necessary that wrapping embryonic tissues be permeable to water. In this sense, biosurfactants, due to their amphipathic structure, probably acted on external wrapping tissue, increasing the permeability of seeds and thereby facilitating germination, as reported by Hameed et al. [69] for surfactants. This property, combined with the high environmental compatibility of these microbial molecules, can lead to the effective application of biosurfactant from *S. marcescens* UCP 1549 in agriculture, reducing the use of agrochemical [70].

### Application of biosurfactant in removal of burned motor oil from contaminated sand

**Fig. 8:**
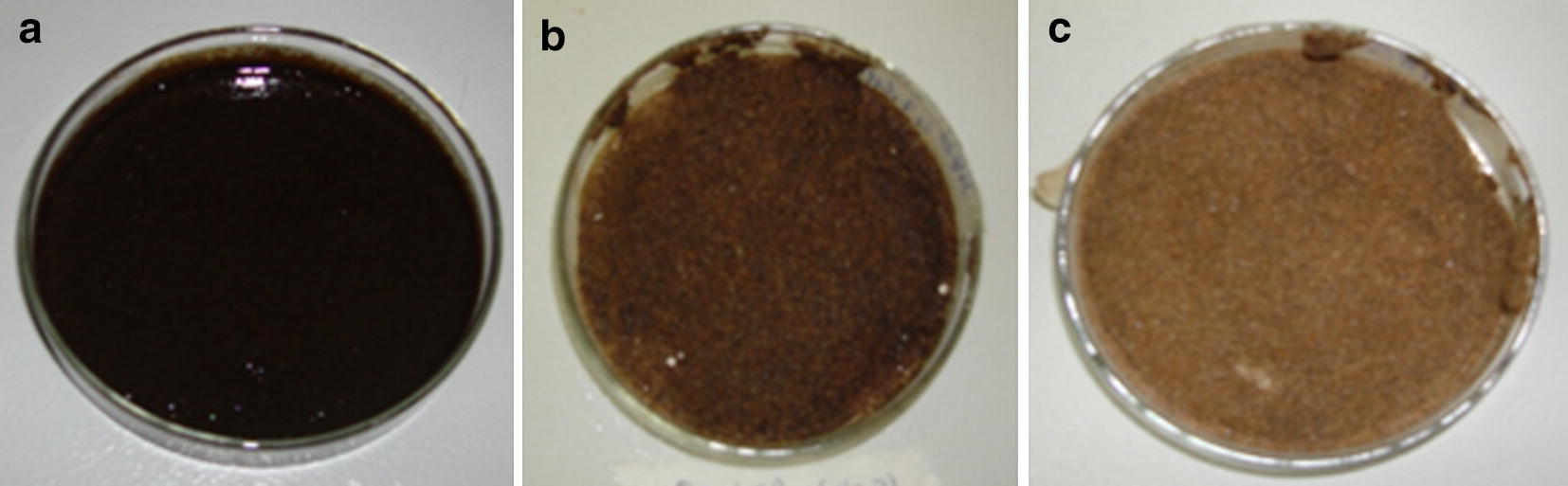
Application of biosurfactant produced by *Serratia marcescens* UCP 1549 in removal of burned motor oil from contaminated sand. Beach sand artificially contaminated with burned motor oil: without treatment (**a**) and after treatment with distilled water (**b**) and biosurfactant produced by *S. marcescens* UCP 1549 (**c**)

Biosurfactants can emulsify hydrocarbons enhancing their water solubility, decreasing surface tension and increasing the displacement of oil substances from soil particles [3, 6, 8, 54]. Results described in the literature show that the biosurfactants produced by strains of *P. aeruginosa* removed 49–54% of crude oil adsorbed in sand [71], whereas Silva et al. [60] demonstrated high removal rates (above 85%) of diesel oil from sand samples, but less than 20% when petroleum was tested. In this study, the crude biosurfactant produced by *S. marcescens* UCP 1549 was able to remove 94% of motor oil contained in sand, while the distilled water (control) removed only 63% (Fig. 8) (Additional file 1: Table S2). Similarly, Nalini and Parthasarathi [55] showed the recovery of 92% of used engine oil adsorbed in sand by biosurfactant from *S. rubidae.*

The effectiveness of biosurfactants produced by others *S. marcescens* strains in oil recovery was previously tested. Anyanwu et al. [26] informed 60% and 51% removal of engine oil and kerosene, respectively, of biosurfactant from *S. marcescens* NSK-1. Biosurfactant of *S. marcescens* UEO15 revealed recoveries of 78% and 59% of crude oil and kerosene, as compared to 10% and 25% obtained by distilled water, respectively [23]. In our previous work, the crude biosurfactant produced by *S. marcescens* UCP 1549, in medium containing only agro-industrial residues, exhibited removing of 88.27% and 73.70% of burned motor oil adsorbed in beach sand and mangrove sediments, respectively [8]. Thus, results obtained here confirmed the considerable potential of these biosurfactants for be applied in bioremediation processes of oil-polluted environments.

## Conclusions

The present study demonstrated the effectiveness of using low-cost medium containing CCW for the production of biosurfactant by *S. marcescens* UCP 1549. The feasibility of utilization of agro-industrial wastes in combination with the application of a full-factorial design proved to be efficient strategies to obtain low-cost biosurfactant and becomes viable its industrial-scale production. The polymeric and non-toxic biomolecule exhibited not only its excellent properties to reduce surface tension, but also its high stability at wide range of pH, temperature and NaCl concentrations. In addition, it showed higher potential for breaking the dormancy in cabbage seeds and removing burned motor oil from beach sand, suggesting its application in agriculture and environmental processes.

## Methods

### Microorganism

*Serratia marcescens* UCP 1549, originally isolated and identified by Araujo et al. [25] was kindly provided by the Culture Collection of the Catholic University of Pernambuco, Recife, Brazil, registered in the World Federation for Culture Collection (WFCC). The bacterium was maintained in Luria-Bertani (LB) solid medium (tryptone 10 g/L, yeast extract 5 g/L, NaCl 10 g/L and agar 15 g/L) at 5 °C. Stored cultures were transferred first to LB medium and incubated for 18 h at 28 °C. Then, two colonies were transferred to 50 mL of LB broth and incubated at 28 °C and 150 rpm in an orbital shaker. Once the optical density at 600 nm reached 0.8–1.0, this culture was used as inoculum.

### Substrates

The substrates used for production of biosurfactant was lactose (Merck), cassava flour wastewater (CWW), kindly supplied by the cassava processing plant located in the municipality of Carnaíba, Pernambuco, Brazil, and corn oil (CO), bough in a local supermarket.

### Biosurfactant production

Biosurfactant production was carried out in 250 mL Erlenmeyer flasks with 100 mL of production medium containing lactose, CWW and CO, according to a full-factorial design (FFD) (“Factorial design” section). The media were adjusted to pH 7.0 and autoclaved at 121 °C for 15 min. Then, they were inoculated at 1% and incubated for 72 h at 28 °C, under orbital agitation (150 rpm).

### Factorial design

In this study, a 2^3^ FFD with 8 assays and 4 replicates at the central point was applied to investigate the effects of each independent variables (lactose, CWW and CO concentrations), as well as the interactions between them, on surface tension as response variable. Table 1 shows the levels studied and the decoded matrix of FFD.

The data obtained from the experiments were subjected to statistical analysis by Statistica^®^ software, version 8.0 (StatSoft Inc., USA) and the significance of the results was tested at *p* < 0.05 level.

### Determination of surface tension

The surface tension was determined on cell-free metabolic liquid obtained by centrifuging (12,000×*g* for 20 min) and filtration of culture media. Analyses were performed at 25 °C in a Sigma 70 tensiometer (KSV Instruments Ltd., Finland), using the Du Nouy ring method [72]. Milli-Q water with surface tension of 72 mN/m was used to calibrate the tensiometer.

### Growth kinetics and biosurfactant production

To determine the growth kinetics, samples were collected every 4 h during the first 12 h, then every 12 h until 120 h of fermentation. Microbial growth was evaluated by “pour plate” technique, inoculating aliquots of 0.1 mL of each sample on Petri plates containing medium LB and incubating at 28 °C for 24 h. Then, each plate was subjected to counting of viable colonies and growth was expressed in CFU/mL. Samples were also subjected to determination of pH and surface tension.

### Studies of biosurfactant stability

Studies of stability of biosurfactant were carried out using 25 mL of cell-free metabolic liquid at different temperatures (0, 5, 28, 70, 100 and 121 °C), for 1 h, and cooled to room temperature, after which the surface tension was measured. The effect of pH on surface tension was evaluated after adjustment of the metabolic liquid pH to 2, 4, 6, 8, 10 and 12 with 2 M HCl or NaOH. The effect of NaCl concentrations (2, 4, 6, 8, 10 and 12%, w/v) on the activity of the biosurfactant were also determined [8].

### Isolation of biosurfactant

The biosurfactant produced by *S. marcescens* UCP 1549 after 72 h of fermentation was isolated from cell-free metabolic liquid obtained by centrifuging (12,000×*g* for 20 min) the culture. The metabolic liquid was subjected to precipitation by adding ammonium sulfate [(NH_4_)_2_SO_4_] salts to achieve 65% of saturation and incubated *overnight* at 4 °C. Then, it was centrifuged at 15,000×*g* for 15 min and the cell-free metabolic liquid was collected and again centrifuged at 5000×*g* for 15 min [73]. The supernatant obtained was discarded and the crude biosurfactant was subjected to dialysis with deionized water for 24 h, with exchanges every 4 h to remove the salt adhered to the sample. The precipitate was collected, lyophilized and used for analysis [54].

### Determination of CMC of biosurfactant

The critical micelle concentration (CMC) of biosurfactant isolated after 72 h of cultivation was determined in aqueous solution. The surface tension was measured on an automatic tensiometer (model Sigma 70 KSV Ltd., Finland) using a platinum-iridium ring. The sample of the isolated biosurfactant was diluted at different concentrations, starting with a minimum concentration of 0.001 mg/mL, until up to reaching a constant value of surface tension. The CMC was seen to have produced a surface tension of a constant value. The value of CMC was obtained by plotting surface tension against percentage concentration of biosurfactant (%) [54].

### Characterization of biosurfactant

#### Chemical composition

The carbohydrate content of the isolated biosurfactant was determined by the method of phenol–sulphuric acid using d-glucose as a standard [74]. The lipids were extracted with chloroform (1:1) three times, after separate the organic phase with the solvent system chloroform/methanol (1:2), the organic phase rotatory evaporator and then weigh the flask, the difference is the amount of lipids [75]. Protein concentrations were evaluated using the kit of total proteins Labtest Diagnostica S.A. (Brazil) for colorimetric determination of total proteins by Biuret reagent.

#### Zeta potential

The electrokinetic of potential zeta was analyzed in the Zeta Meter instrument using 100 mg of biosurfactant in 5 mL of an aqueous solution of KCl, with the corresponding ionic strength at 0.001 M [54].

#### Gel chromatography

Elution was performed using the PD10 column (Sephadex G25) column XK 16/40 GE Healthcare. Initially, the column was equilibrated with 25 mL of distilled water and then applied 2 mL of sample containing high concentrations of salt. After the entry of the sample, the elution of the biopolymer was made with distilled water.

#### Fourier transform infrared spectroscopy (FT-IR)

The identification of functional groups in the isolated biosurfactant was carried out by Fourier-transform infrared spectroscopy (FTIR) on Bruker IFS 66 spectrometer, using KBr pellets. The spectrum was generated in the wavelength range of 4000–400 cm^−1^.

### Determination of biosurfactant phytotoxicity

The phytotoxicity of the biosurfactant was evaluated in seeds of cabbage (*Brassica oleracea*) according to Tiquia et al. [61]. The biosurfactant isolated was tested at concentrations of 10, 50, 125, 250, 500 and 1000 mg/L. 5 mL of each biosurfactant solution were inoculated into Petri plates containing ten disinfected seeds under sterilized Whatman no. 1 filter paper. The plates were incubated for 120 h at room temperature in the dark. The tests were performed in triplicate and it was used distillated water for the control test [76]. Phytotoxicity of biosurfactant was determined by seed germination, root elongation (≥ 5 mm) and germination index (GI, a factor of relative seed germination and relative root elongation), according to the following equations:

$${\text{Relative}}\;{\text{seed}}\;{\text{germination}}\left( \% \right) = \left( {{\text{number}}\;{\text{of}}\;{\text{seeds}}\;{\text{germinated}}\;{\text{in}}\;{\text{the}}\;{\text{extract}}/{\text{number}}\;{\text{of}}\;{\text{seeds}}\;{\text{germinated}}\;{\text{in}}\;{\text{the}}\;{\text{control}}} \right) \times 100.$$

$${\text{Relative}}\;{\text{root}}\;{\text{length}}\left( \% \right) = \left( {{\text{mean}}\;{\text{root}}\;{\text{length}}\;{\text{in}}\;{\text{the}}\;{\text{extract}}/{\text{mean}}\;{\text{root}}\;{\text{length}}\;{\text{in}}\;{\text{the}}\;{\text{control}}} \right) \times 100$$

$${\text{Germination}}\;{\text{index}} = \left[ {\left( {\% \;{\text{of}}\;{\text{seed}}\;{\text{germination}}} \right) \times \left( {\% \;{\text{of}}\;{\text{root}}\;{\text{growth}}} \right)} \right]/100\% .$$

### Application of biosurfactant in removal of hydrophobic pollutant in sand

Biosurfactant suitability for removing hydrophobic pollutants was determined using artificially contaminated sand containing 5% burned motor oil. Twenty-gram samples of contaminated sand were transferred to 250 Erlenmeyer flask and submitted to the following treatments: (A) addition of 50 mL of distilled water (control), (B) addition of 50 mL of cell-free metabolic liquid and (C) addition of 50 mL of aqueous solution of the isolated biosurfactant at concentration of 1000 mg/L. The flasks were subjected to 150 rpm for 48 h at 28 °C and then, centrifuged at 5000×*g* for 20 min for separation of the washing solution and sand sediment. The amount of oil remaining in the sand was gravimetrically determined by hexane [8, 77].

## Additional file

**Additional file 1: Table S1.** Average of chemical composition of cassava wastewater. **Table S2.** Application of biosurfactant produced by *Serratia marcescens* UCP 1549 in removal of burned motor oil from contaminated marine soil. **Figure S1.** Growth profile of *Serratia marcescens* UCP 1549 on Luria Bertani (LB) medium, during 144 h at 28 °C and 150 rpm.
