# Supplementary material for: Sustainable biosurfactant produced by Serratia marcescens UCP 1549 and its suitability for agricultural and marine bioremediation applications
Source: Microb Cell Fact. 2019 Jan 4;18:2. doi: 10.1186/s12934-018-1046-0 (PMC6318876; doi:10.1186/s12934-018-1046-0)
Supplement: Supplementary file 1 — Additional file 1: Table S1. Average of chemical composition of cassava wastewater. Table S2. Application of biosurfactant produced by Serratia marcescens UCP 1549 in removal of burned motor oil from contaminated marine soil. Figure S1. Growth profile of Serratia marcescens UCP 1549 on Luria Bertani (LB) medium, during 144 h at 28 °C and 150 rpm. [file 12934_2018_1046_MOESM1_ESM.docx]

**Additional file 1**

**Table S1.** Average of chemical composition of cassava wastewater.

| **Parameters** | **References** | | | | | |
| --- | --- | --- | --- | --- | --- | --- |
|  | **da Silva et al. (2005)** | **Nitschke and Pastore (2006)** | **Costa et al. (2009)** | **de Oliveira et al. (2013)** | **Xie et al. (2014)** | **dos Santos et al. (2017)** |
| Total sugars (g/L) |  | 35.3 | 56.4 |  | 5.2 |  |
| Reducing sugars (g/L) |  | 12.8 |  | 0.57 |  |  |
| Non-reducing sugars (g/L) |  | 22.2 |  |  |  |  |
| Total nitrogen (g/L) | 0.032 | 2.5 |  | 0.16 | 0.24 | 0.098 |
| Phosphorus (mg/L) | 17.8 | 225.9 | 900 |  |  |  |
| Potassium (mg/L) | 333.6 | 2665.1 | 3600 |  |  |  |
| Calcium (mg/L) | 31.37 | 272.5 |  |  |  |  |
| Magnesium (mg/L) | 36.87 | 519.0 | 500.0 |  |  |  |
| Sulfur (mg/L) |  | 104.0 |  |  |  |  |
| Iron (mg/L) | 6.9 | 7.8 | 6.1 |  |  |  |
| Zinc (mg/L) | 0.59 | 7.3 | 11.1 |  |  |  |
| Manganese (mg/L) | 0.62 | 1.8 | 4.1 |  |  |  |
| Cooper (mg/L) | 0.05 | 0.6 | 14.1 |  |  |  |
| pH | 4.8 | 5.9 |  | 5.1 | 4 | 6.8 |
| Soluble COD (g O_2_/L) |  |  |  |  | 19.4 |  |
| COD (g O_2_/L) | 14.7 | 55.82 |  | 16.09 |  | 6.8 |
| NH_4_ (mg/L) |  |  |  | 14.4 | 8 |  |
| Cyanide (mg/L) | 12 |  |  |  |  | 1.9 |

COD: chemical oxygen demand

**Table S2.** Application of biosurfactant produced by *Serratia marcescens* UCP 1549 in removal of burned motor oil from contaminated marine soil.

| **Treatments** | **Removed oil (%)** | **Remaining oil (%)** |
| --- | --- | --- |
| Distilled water (control) | 63 | 37 |
| Metabolic liquid containing the biosurfactant | 94 | 6 |

**Figure S1.** Growth profile of *Serratia marcescens* UCP 1549 on Luria Bertani (LB) medium, during 144 h at 28ºC and 150 rpm.


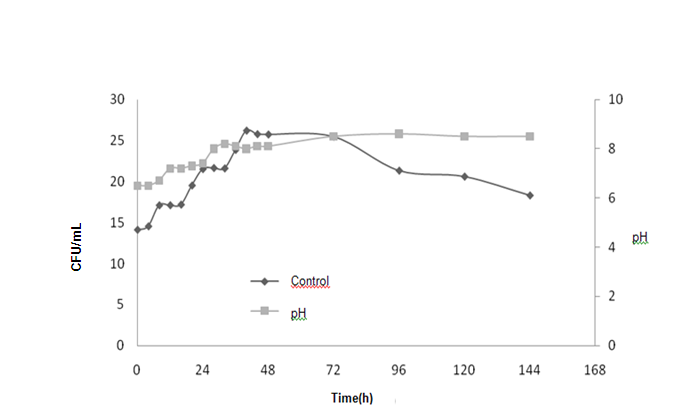


**References**

da Silva FF, Bertonha A, Freitas PSL, Muniz AS, Rezende R, Gonçalves ACA, Labegalini L, Neto VSC. Desdobramento do amido em glicose para identificar água residuária de residuária de indústria indústria indústria de mandioca no perfil do solo de mandioca no perfil do solo de mandioca no perfil do solo. Acta Sci. Biol Sci 2005;27, 507-511.

Nitschke M, Pastore, GM. Production and properties of a surfactant obtained from *Bacillus subtilis* grown on cassava wastewater. Biores Technol 2006;97 <https://doi.org/10.1016/j.biortech.2005.02.044>

Costa SG, Lépine F, Milot S, Déziel E, Nitschke M, Contiero J. Cassava
wastewater as a substrate for the simultaneous production of rhamnolipids and
polyhydroxyalkanoates by *Pseudomonas aeruginosa*. J Ind Microbiol Biotechnol. 2009; 36, <https://doi.org/10.1007/s10295-009-0590-3>

de Oliveira SMM, Gomes SD, Sene L, Machado SR, Coelho ACB, Cereda MP, Christ D, Piechontcoski J. Production of 2-phenylethanol by *Geotrichum fragrans*, *Saccharomyces cerevisiae* and *Kluyveromyces marxianus* in cassava wastewater. J Food Agric Env 2013;11, 158–163.

Xie L, Liu H, Chen Y, Zhou Q. pH-adjustment strategy for volatile fatty acid production from high-strength wastewater for biological nutrient removal. Water Sci Technol 2014;69 <https://doi.org/10.2166/wst.2014.120>

dos Santos JD, Veit MT, Palácio SM, da Cunha Gonçalves G, Fagundes-Klen MR. Evaluation of the combined process of coagulation/flocculation and microfiltration of cassava starch wastewater: removal efficiency and membrane fouling. Water Air Soil Pollut. 2017;228. <https://doi.org/10.1007/s11270-017-3416-3>
